# Prelimbic cortex maintains attention to category-relevant information and flexibly updates category representations

**DOI:** 10.1016/j.nlm.2021.107524

**Published:** 2021-11

**Authors:** Matthew B. Broschard, Jangjin Kim, Bradley C. Love, Edward A. Wasserman, John H. Freeman

**Affiliations:** a Department of Psychological and Brain Sciences, University of Iowa, Iowa City, IA 52242, USA; b Department of Experimental Psychology and The Alan Turing Institute, University College London, London, UK

**Keywords:** Prelimbic prefrontal cortex, Rat, Category learning, Executive functions, SUSTAIN, Touchscreen

## Abstract

Category learning groups stimuli according to similarity or function. This involves finding and attending to stimulus features that reliably inform category membership. Although many of the neural mechanisms underlying categorization remain elusive, models of human category learning posit that prefrontal cortex plays a substantial role. Here, we investigated the role of the prelimbic cortex (PL) in rat visual category learning by administering excitotoxic lesions before category training and then evaluating the effects of the lesions with computational modeling. Using a touchscreen apparatus, rats (female and male) learned to categorize distributions of category stimuli that varied along two continuous dimensions. For some rats, categorizing the stimuli encouraged selective attention towards a single stimulus dimension (i.e., 1D tasks). For other rats, categorizing the stimuli required divided attention towards both stimulus dimensions (i.e., 2D tasks). Testing sessions then examined generalization to novel exemplars. PL lesions impaired learning and generalization for the 1D tasks, but not the 2D tasks. Then, a neural network was fit to the behavioral data to examine how the lesions affected categorization. The results suggest that the PL facilitates category learning by maintaining attention to category-relevant information and updating category representations.

## Highlights

• Rats categorize distribution of stimuli containing two continuous dimensions.
• Prefrontal lesions impair category tasks containing irrelevant stimulus information.
• Prefrontal lesions do not affect tasks containing only relevant stimulus information.
• Prefrontal lesions impair trial-by-trial updating of category representations.

## 1 Introduction

Categorization is the process of grouping perceptually or functionally related objects and events. Abundant evidence from neuroimaging (Kumaran et al., 2009, Bowman and Zeithamova, 2018) and physiology (Freedman, 2001) experiments supports the recruitment of prefrontal cortex (PFC) in categorization tasks. The PFC is also important for transitive inference, a mechanism that infers new information and promotes generalization by extrapolating overlapping information across multiple episodes (Koscik and Tranel, 2012, Zeithamova et al., 2012).

**Fig. 1:**
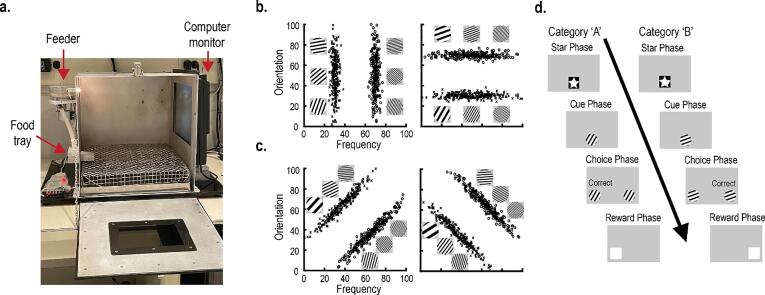
**A,** Behavioral testing was conducted in custom-built chambers. Each chamber contained a computer monitor and a touchscreen panel so that the rats could interact with the visual stimuli. A feeder delivered food pellets into a food tray to reinforce behavior. **B-C,** Rats were randomly assigned to learn one of four category tasks. For each task, category exemplars contained gratings that varied in their spatial frequency and orientation. Categories were created by placing normal distributions on this two-dimensional stimulus space. **B**, For the 1D tasks, category distributions were perpendicular to a stimulus axis. Consequently, one stimulus dimension was category-relevant (i.e., the dimension perpendicular to the distributions); the second dimension was category-irrelevant. We predicted that would rats use selective attention to learn 1D tasks by shifting attention towards the relevant dimension. **B,** For the 2D tasks, category distributions were not perpendicular to a stimulus axis. Therefore, both stimulus dimensions were category-relevant. **C,** The typical trial sequence for all training and testing sessions. Rats initiated each trial by touching the star stimulus at the center of the screen (Star phase). Then, an exemplar was randomly generated from the category distributions and placed at the center of the screen (Cue phase). The rat touched this exemplar three times, at which point copies of the exemplar were presented at the left and right sides of the screen (Choice phase). These copies acted as report keys. Members of category ‘A’ required a touch to the left report key, and members of category ‘B’ required a touch to the right report key. For correct responses, a white box appeared on the screen (Reward phase); one touch of the white box delivered a food reward. For incorrect responses, a correction trial was initiated, where the trial repeated from the Cue phase after a timeout.

Accordingly, theories of categorization predict that the PFC plays a substantial role in learning new categories. COVIS (COmpetition between Verbal and Implicit Systems) posits that the PFC governs a declarative system that learns new categories by testing explicit category rules (Ashby et al., 1998). The COVIS framework has been tested empirically by training participants to categorize distributions of visual stimuli that vary along two continuous dimensions (Maddox et al., 2003, Smith et al., 2012). In one condition, only one stimulus dimension is category-relevant, and learning involves *selective attention* to that dimension (1D tasks; Fig. 1B). In a second condition, both stimulus dimensions are relevant, and learning requires *divided attention* to both dimensions (2D tasks; Fig. 1C). COVIS predicts that the declarative system (and the PFC) is important for learning 1D tasks, as they can be solved by a unidimensional category rule (Ashby & Maddox, 2011). This prediction is supported by neuroimaging experiments (Nomura et al., 2006).

**Fig. 2:**
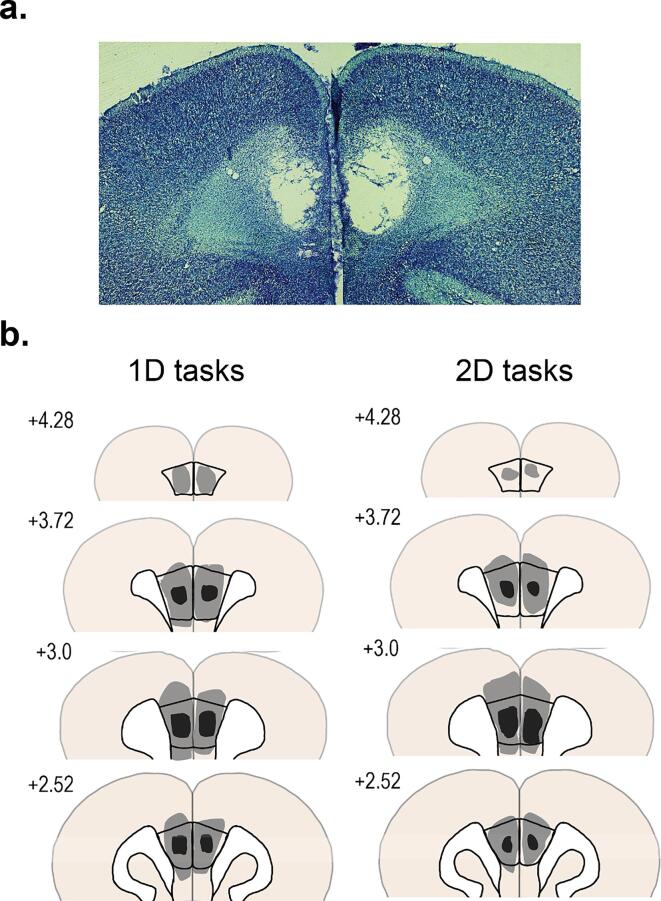
**A,** A representative example of the location and spread of the PL lesions. **B,** A comparison of lesion size and location for the smallest lesion (light gray) and the largest lesion (dark gray) for rats learning a 1D task (left) and rats learning a 2D task (right). All lesions were centered in the PL and were contained within bregma + 4.3 and + 2.2. Lesions rarely extended into cingulate cortex and infralimbic cortex.

**Fig. 3:**
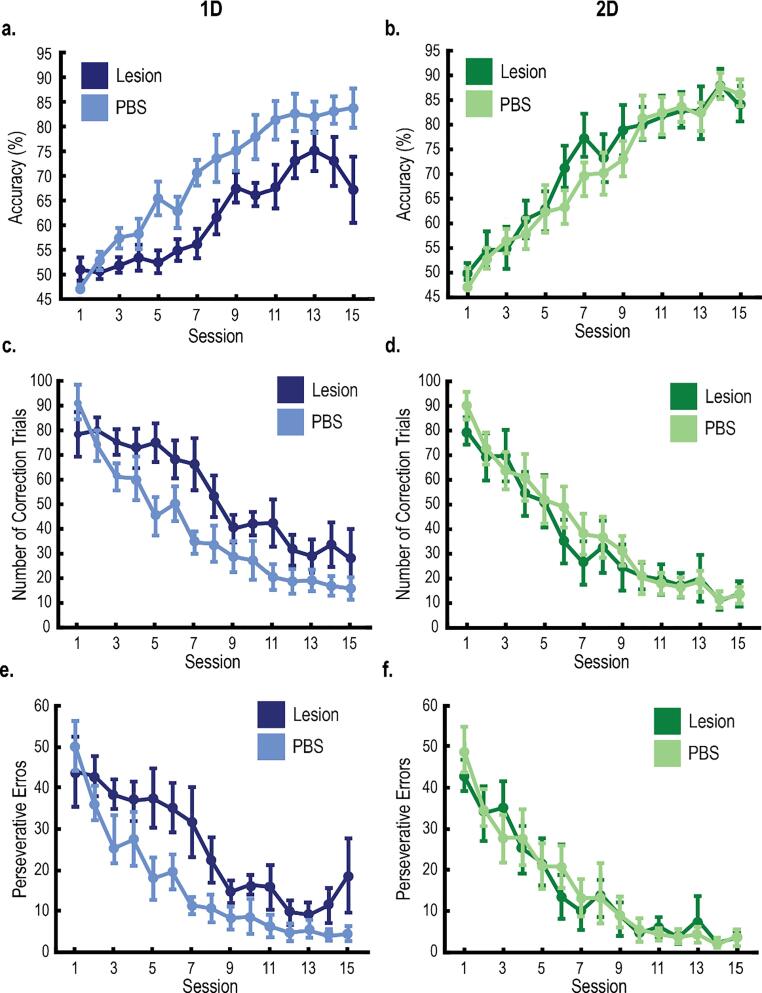
Excitotoxic lesions of the PL impaired learning 1D tasks, but not 2D tasks. **A-B,** Mean session accuracy of rats learning 1D tasks (A) and 2D tasks (B) (n = 8 per group). Compared to controls, rats with PL lesions had impaired accuracy for 1D tasks, but not for 2D tasks. Impairments were greatest at the beginning of category training. **C-D,** Mean number of correction trials from rats learning 1D tasks (C) and 2D tasks (D). Compared to controls, rats with PL lesions learning the 1D tasks, but not the 2D tasks required more correction trials. **E-F,** Mean number of perseverative errors for rats learning the 1D tasks (E) and 2D tasks (F). Compared to controls, rats with PL lesions learning the 1D tasks, but not the 2D tasks made more perseverative errors, where a choice was repeated after receiving negative feedback. All error bars indicate the *SEM*.

Rodents have become great models to examine mechanisms underlying complex behavior (Zoccolan et al., 2009, Vinken et al., 2014). We recently developed rodent versions of the 1D and 2D tasks using a touchscreen apparatus to investigate rat category learning (Broschard, Kim, Love, Wasserman, & Freeman, 2019). The current experiment extends this work by examining the contributions of the prelimbic (PL) area of the rat PFC. Broschard et al., 2019 concluded that rats use selective attention to learn the 1D tasks and bias attention towards the category-relevant dimension. We predict that this is mediated by the PL; therefore, inactivating the PL will impair learning for the 1D tasks. This prediction is supported by calcium imaging in the mouse medial frontal cortex during a go/no-go version of the 1D task (Reinert et al., 2021). This prediction also aligns with Love & Gureckis (2007), who proposed that the PFC is synonymous to the selective attention mechanism of the neural network model SUSTAIN (Supervised and Unsupervised STratified Adaptive Incremental Network; Love, Medin, & Gureckis, 2004). The current experiment tested this prediction directly.

There is contention regarding whether rodent PL is comparable to the primate PFC (Laubach, Amarante, Swanson, & White, 2018). PL satisfies early definitions of PFC by exhibiting bidirectional communication with the medial dorsal thalamus (Rose & Woolsey, 1948). Additionally, some functions of PL are analogous to primate PFC, including working memory (Horst & Laubach, 2009), goal directed behavior (Ostlund 2005), response conflict (Wit, Kosaki, Balleine, & Dickinson, 2006), behavioral flexibility (Ragozzino 2007), and attention (Tait, Bowman, Neuwirth & Brown, 2018). However, anatomical investigations conclude that PL may be homologous to cingulate cortex in primates (Heilbronner et al., 2016). Furthermore, all of rodent frontal cortex is agranular, highlighting large differences in the cellular makeup between rodents and primates (Uylings and Eden, 1991, Seamans et al., 2008). Therefore, generalizing the results of the current experiment to primate PFC requires careful consideration of anatomical and functional comparisons.

Here, we investigated the role of the PL in visual category learning in rats. Rats underwent stereotaxic surgery to lesion the PL with NMDA. After recovery, the rats were trained to learn the 1D or 2D categorization tasks. Then, we fit the neural network SUSTAIN to the behavioral data to further examine the role of the PL, specifically as it pertains to selective attention. Together, the results suggest that the PL maintains attention to category-relevant information and updates category representations according to recent exemplars.

## 2 Materials and methods

### 2.1 Subjects

Male (n = 16, mean weight: ∼350 g) and female (n = 16, mean weight: ∼250 g) Long-Evans rats were studied. Upon arriving in the animal colony, rats were put on a 12-hour light/dark cycle and given *ad libitum* access to food and water. After acclimating to the new environment for a week, food was restricted. Weights were recorded daily to ensure weights did not go below 85% of the rats’ free feeding weight. All procedures were approved by the Institutional Animal Care and Use Committee at the University of Iowa.

### 2.2 Touchscreen apparatus

For all experimental sessions, rats were placed within custom-built touchscreen chambers (Fig. 1A; 36 × 41 × 36 cm). The chambers contained a computer monitor (Model 1550 V, NEC, Melville, NY) mounted on one wall to present visual stimuli to the rats. A touchscreen (15-in, Elo Touch Systems, Fremont, CA) was placed in front of the computer monitor so that the rats could interact with the screen. On the wall opposite from the monitor, a food tray (6.5 × 13 × 4.5 cm) delivered food pellets to the rat via a rotary pellet dispenser (Med Associates Inc., Georgia, VT, model ENV-203IR) that was controlled by an electrical board (Model RS-232, National Control Devices, Osceola, MO). A house light above the food tray was always on during experimental sessions. White noise within the room was also always on to minimize distractions. Custom MATLAB scripts controlled all experimental sessions and procedures (MathWorks, Natick, MA). Finally, a camera (model ELP-USB100W05MT-RL36) was mounted to the ceiling of the chamber and faced the computer screen so that the rats’ behavior could be observed and recorded.

**Fig. 4:**
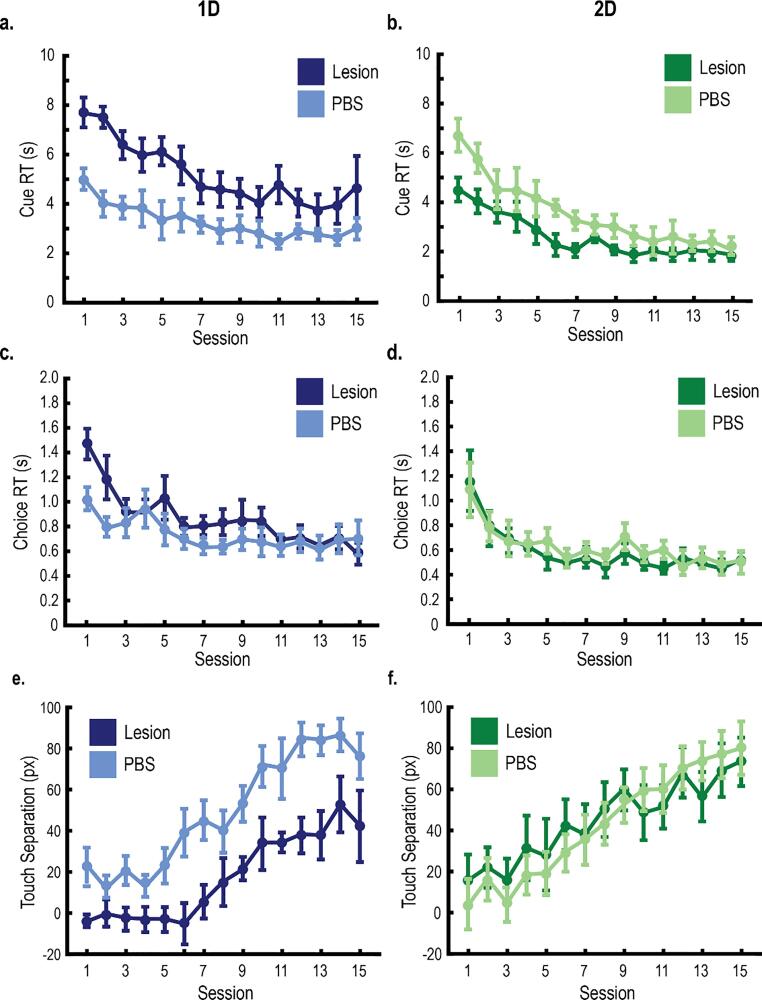
Excitotoxic lesions of the PL affected reaction time and choice anticipation during category learning. **A-B,** Mean time to observe and categorize each exemplar (Cue RT) for rats learning 1D tasks (A) and 2D tasks (B). Compared to controls, rats with PL lesions learning the 1D tasks, but not the 2D tasks exhibited a longer Cue RT. **C-D,** Mean time to execute a category decision (Choice RT) for rats learning the 1D tasks (C) and 2D tasks (D). Compared to controls, PL lesions did not affect Choice RT. **E-F,** Touch separation used the x-coordinate of the three touches during the Cue phase to estimate choice confidence. Positive touch separation indicates horizontal movement of the rat towards the correct side, whereas negative touch separation indicates horizontal movement towards the incorrect side. Compared to controls, rats with PL lesions learning the 1D tasks (A), but not the 2D tasks (B) exhibited lower touch separation across category learning. All error bars indicate the *SEM*.

### 2.3 Pre-training procedures

Once food restriction began, each rat was handled daily for 1 week. This reduced the stress of interacting with experimenters. Then, each rat underwent cart training, which encouraged the foraging of food pellets in an open field. Each rat was placed on the surface of a laboratory cart, and twenty 45-mg pellets were scattered on the cart’s surface. This procedure was repeated daily until the rat consumed all pellets within 15 min, which usually took about 7 days. After cart training, rats underwent a daily shaping procedure to learn to interact with the touchscreen (Broschard, Kim, Love, & Freeman, 2020). This procedure included three separate phases; each phase was incrementally similar to the trial sequence used during training and testing sessions. Phase I required a minimal touch requirement and was used to orient the rats to the screen. Each trial began with the presentation of a star at the center of the screen. After 15 s (or one touch of the screen), the star was replaced by a white box appearing on the left or right side of the screen. A food pellet was delivered if the rat touched anywhere on the screen while the white box was presented. Otherwise, the trial aborted after 45 s, and the trial was considered a miss. This procedure was repeated until the rat completed at least 55/60 trials within 25 min. In Phase II, the touch requirement was increased. Specifically, the rats were required to touch both the star stimulus and the white box to receive a food reward. Similar to Phase I, the trial phases timed out (i.e., 15 s for the star stimulus and 45 s for the white box) in the absence of a response. Sessions continued until the rat completed at least 55 trials within 30 min. Phase III was identical to Phase II except that the trials did not time out. Sessions continued until the rat completed all 60 trials within 25 min. All shaping procedures required about 14 days.

### 2.4 Surgery

After shaping was complete, rats underwent stereotaxic surgery. Under isoflourane (1% − 4%) anesthesia), a Hamilton syringe (1 uL; 26 gauge) was lowered into the PL bilaterally (AP: +3.0; ML: ±0.7; DV; −3.5). Upon reaching the target site, 0.4 µL of either NMDA (20 mg/ml; 10 µL/h; Sigma-Aldrich, St. Louis, MO) or PBS was infused. After surgery, rats were placed on a heating pad until awake and mobile to prevent hypothermia. Meloxicam (1 mg/ml) was administered as analgesic both during surgery and 24 h after surgery. Rats were allowed at least one week to recover.

### 2.5 Behavioral testing: An overview

After a week of recovery, rats were given multiple training and testing sessions to learn to categorize visual stimuli. Briefly, on each trial, a single stimulus appeared on the screen, and the rat decided its category membership (i.e., category ‘A’ or category ‘B) by pressing one of two report keys (Fig. 1D). Food reinforcement was delivered after correct responses to guide learning.

### 2.6 Category stimuli

The category stimuli (239 × 239 pixels) presented to the rats contained black and white gratings (Fig. 1B-D). Across stimuli, these gratings varied along two continuous dimensions: spatial frequency and orientation. The spatial frequency of the gratings ranged from 0.2532 cycles per visual degree (cpd) to 1.2232 cpd, and the orientation of the gratings ranged from 0 rad to 1.75 rad. These values were obtained from pilot experiments and are within the perceptual limits of rats (Crijns & Op de Beeck, 2019). Linear transformations of these dimensions were made so that both dimensions had a common range (i.e., 0 to 100). Specifically,

$$Normalized\;frequency=\frac{\mathrm{cpd}}{0.0097}-26.10$$

$$Normalized\;orientation=radians*\frac{180}{\mathrm{pi}}$$

A two-dimensional stimulus space was created using these transformed stimulus dimensions (Fig. 1B-C).

### 2.7 Category tasks

Category tasks were created by placing bivariate normal distributions on this transformed stimulus space (Fig. 1B; Category A: *µ_X_* = 30, *σ_X_* = 2.5, *µ_Y_* = 50, *σ_Y_* = 20; Category B: *µ_X_* = 70, *σ_X_* = 2.5, *µ_Y_* = 50, *σ_Y_* = 20; Broschard et al., 2019, Broschard et al., 2020, O’Donoghue et al., 2020). Each distribution constituted a category, and each point within a distribution represented a category stimulus. Three additional category tasks were created by rotating these distributions in 45-degree increments (Fig. 1B-C). Importantly, rotating the distributions did not affect any physical properties of the distributions (Ashby, Smith, & Rosedahl, 2019; e.g., standard deviation, mean between-category distance, etc.). However, these rotations changed how the distributions were oriented in relation to the axes of the stimulus space. 1D tasks had distributions that were perpendicular to one of the stimulus dimensions (Fig. 1B). Because of this orientation, only one dimension (i.e., the perpendicular dimension) was category-relevant and had to be considered when deciding category membership. The dimension parallel to the distributions was category-irrelevant and could be ignored. Conversely, 2D tasks had distributions that were not aligned with either stimulus axis (Fig. 1C). For these tasks, both dimensions were category-relevant, and deciding category membership involved combining information from both dimensions.

### 2.8 Category training

Rats were randomly assigned to learn one of the four category tasks (Broschard et al., 2019, Broschard et al., 2020). Rats were given 15 training sessions; each session contained 80 training trials. On each trial, a star stimulus was presented at the center of the screen (Fig. 1D; Star Phase). After one touch of the star, a category exemplar was randomly selected from the training distributions (Fig. 1B-C) and replaced the star stimulus (Cue Phase). After three touches of this exemplar, copies of the exemplar were presented on the left and right sides of the screen, acting as report keys (Choice Phase). Rats touched either report key depending on the category membership of the exemplar. The categories were mapped spatially, such that the left report key was chosen for members of category A, and the right report key was chosen for members of category B. If the correct side was chosen, a white box replaced the report key (Reward Phase). One touch of the white box delivered a food reward. If instead the incorrect side was chosen, then a correction trial was initiated. Here, the trial repeated from the Cue Phase after a 5 to 10 s time-out. Correction trials were repeated without reinforcement until the correct side was chosen. Inter-trial intervals ranged from 5 to 10 s.

### 2.9 Category generalization

**Fig. 5:**
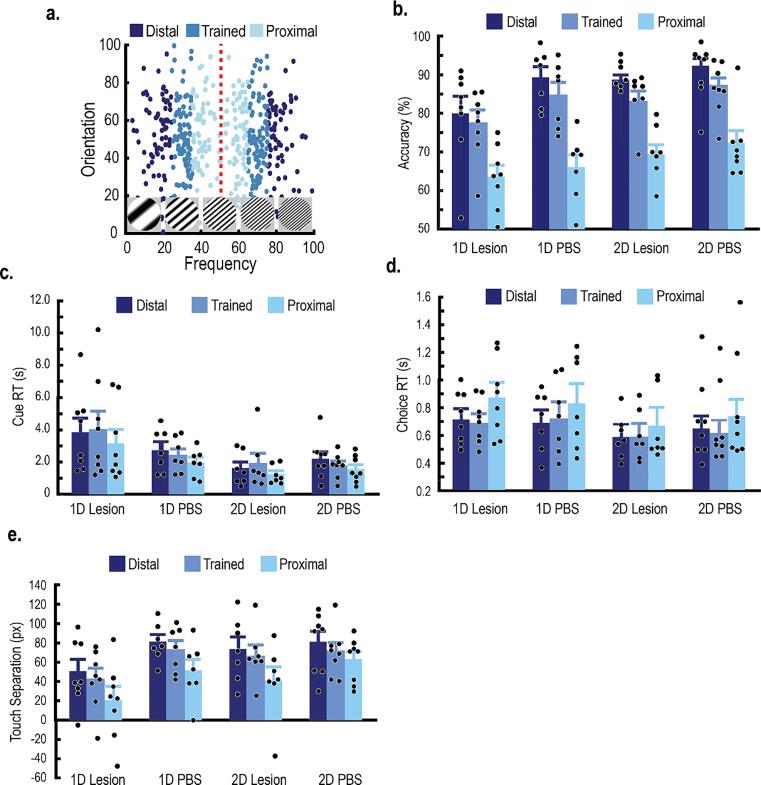
The PL lesions impaired category generalization in rats trained on the 1D tasks, but not the 2D tasks. **A,** Each rat was given five testing sessions to examine category generalization. Testing distributions had the same category means as the training distributions, but the standard deviation along the relevant dimension was expanded to cover novel portions of the stimulus space. Each dot within the distributions represents a unique Gabor patch presented during testing. Testing distributions were split into three trial types: exemplars that overlapped with the training distributions (Trained), novel exemplars closer to the category boundary (Proximal), and novel exemplars farther from the category boundary (Distal). **B,** Mean accuracy across trial types. Generally, accuracy increased according to the distance from the category boundary. PL lesions impaired generalization in rats that learned the 1D tasks, but not rats that learned the 2D tasks. **C,** Mean Cue RT across trial types. Cue RT was larger for rats with PL lesions and had learned the 1D tasks than all other groups. There were no significant interactions across trial types. **D,** Mean Choice RT across trial types. Generally, Choice RT was larger for Proximal trials. The PL lesions did not affect Choice RT. **E,** Mean touch separation across trial types. Touch separation was reduced for rats with PL lesions that learned the 1D tasks. There were no significant interactions across trial types. All error bars indicate the *SEM*.

After category training, rats were presented with five testing sessions to examine category generalization (Broschard et al., 2019, Broschard et al., 2020). Each session contained 80 trials. The trial sequence was identical to training sessions except that correction trials were not administered after incorrect responses (and therefore all choices were reinforced). Exemplars were randomly sampled from testing distributions (Fig. 5A). Testing distributions were identical to the training distributions, except that the standard deviation along the relevant dimension (or axis for the 2D tasks) was increased (*σ_X_* = 10; Broschard et al., 2019, O’Donoghue et al., 2020). With this manipulation, some exemplars overlapped with the training distributions (i.e., Trained; within two standard deviations), but some exemplars sampled from novel portions of the stimulus space. Among the novel exemplars, about half were closer to the category boundary than the training distributions (Proximal), and half were farther from the category boundary (Distal). Generalization to the novel stimuli ensures that the rats did not simply memorize single exemplars during training.

### 2.10 Simple discrimination

**Fig. 6:**
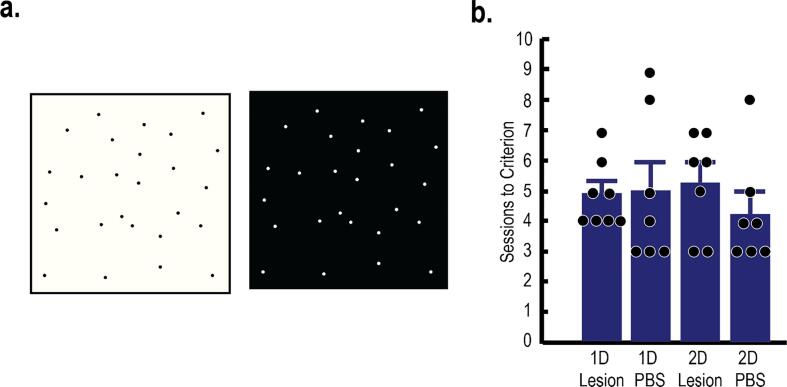
Rats were presented training sessions to learn to discriminate a dark box from a light box. All groups reached learning criterion (75% accuracy for both stimuli) in an equal number of training sessions. All error bars indicate the *SEM*.

After category testing, rats were trained to learn a simple discrimination task. This acted as a control task to ensure that any differences across groups were not caused by deficits in movement, motivation, perception, etc. Instead of categories of stimuli, only two images were presented during training sessions (i.e., a light box and a dark box; Fig. 6A; Kim, Castro, Wasserman, & Freeman, 2018). Both images contained a common pattern of dots to add perceptual complexity. The trial sequence was identical to categorization sessions. The white stimulus was mapped to the left report key, and the black stimulus was mapped to the right report key. Each session contained 72 training trials. Sessions continued until the rat reached a learning criterion (i.e., at least 75% accuracy for both images on two consecutive sessions).

### 2.11 Histology

After all behavioral testing, rats were perfused to verify lesion placements. Rats were given a lethal dose of euthanasia solution (sodium pentobarbital) and then perfused with ∼ 400 mL PBS and ∼ 400 mL of 10% formalin. Brains were stored at 4° C in a solution containing 10% formalin and 30% sucrose. A sliding microtome collected 50 µm coronal sections of the target area. Brain sections were then stained with thionin (Sigma-Aldrich, St. Louis, MO). A close investigation of the tissue was conducted under a light microscope to characterize the size of each lesion within the PL and how much it extended dorsally and ventrally. The boundary of the PL was defined according to Paxinos and Watson (1998).

### 2.12 Statistical analysis

Multiple dependent measures quantified performance for training and testing sessions. First, session accuracy was defined as the proportion of correct responses during the Choice phase. Second, perseverative errors were calculated and were defined as a repeated incorrect response after receiving negative feedback. Third, reaction time was calculated during the Cue phase and Choice phase to quantify the amount of time to 1) observe the stimulus and 2) make a category decision. Reaction times from incorrect trials were excluded from all analyses. Additionally, reaction times that exceeded two standard deviations of the mean were excluded from all analyses, a criterion that is commonly used to eliminate outliers (O’Donoghue et al., 2020). These outliers rarely occurred. Fourth, touch separation used the pixel location of touches during the Cue phase of correct trials to quantify choice confidence. Prior experiments demonstrated that as accuracy improves, the x-coordinate of touches during the Cue phase deviate towards the correct side in anticipation of the rats’ choice (Kim, Castro, Wasserman, & Freeman, 2018). Touch separation is calculated by comparing the x-coordinate of a touch to the average x-coordinate of all three touches from that trial. Positive touch separation indicates deviation towards the correct side, and negative touch separation indicates deviation towards the incorrect side.

These dependent measures were analyzed using linear mixed effects modeling (R, version 3.4.2). Models used for training sessions included fixed effects for experimental group, training session, and a quadratic function across training sessions, as well as random effects for slope, intercept, and the quadratic function. Models for testing sessions included fixed effects for experimental group, trial type (Distal, Trained, and Proximal), and a quadratic function across trial types, as well as random effects for slope, intercept, and the quadratic function. Quadratic functions were used because they best fit the data, and higher order terms did not significantly improve these fits. Sex was added as a covariate for all models to check whether there were any significant differences between male and female rats. To find the simplest model that fit the data, we used a model simplification strategy (Crawley 2007). We started with the full model and then systematically removed random effects one at a time. This continued until the estimates were significantly different from the larger model before it.

### 2.13 SUSTAIN model fitting

SUSTAIN is a neural network model of human category learning and has been used in multiple contexts to map neural activity to specific cognitive processes (e.g., Love and Gureckis, 2007, Mack et al., 2016). Here, we used SUSTAIN to further examine the role of the PL by simulating the effects of the PL lesions on category learning. We were particularly interested whether the PL serves a function similar to SUSTAIN’s attention mechanism (Love & Gureckis, 2007).

**Fig. 7:**
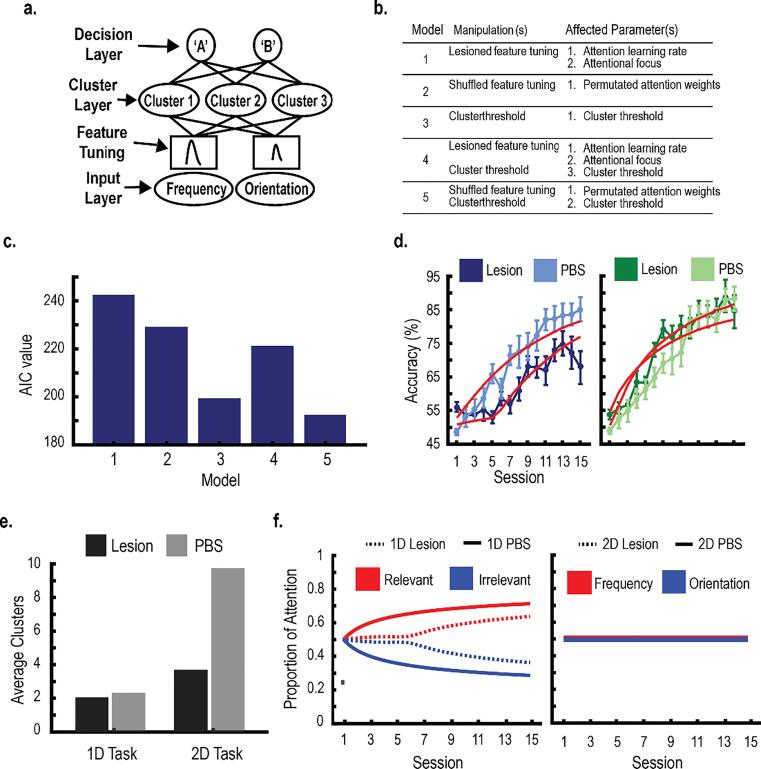
**A,** A diagram of the neural network model SUSTAIN, which contains three distinct layers: the input layer, cluster layer, and decision layer. SUSTAIN also contains a mechanism of selective attention (i.e., the feature tuning mechanism) that weights stimulus information according to category relevance. **B,** Descriptions of the five SUSTAIN models that were fit to the learning data to test the effects of the PL lesions on category learning. These models were compared to a control model which assumed the lesions had no effect on learning. **C,** The best fitting model was determined by comparing the estimated AIC values. The model that best fit the data (Model 5) assumed that the PL maintains attention to category-relevant information and updates category representations. All models produced a better fit than the control model that assumed the lesions had no effect on learning (not graphed: AIC = 278). **D,** SUSTAIN’s predictions using the best fitting model for rats learning the 1D (left) and 2D tasks (right). All error bars indicate the *SEM*. **E,** Mean number of clusters recruited by SUSTAIN using the best fitting model. Generally, SUSTAIN recruited two clusters (one per category) to learn the 1D tasks and multiple clusters (3–4 per category) to learn the 2D tasks. For the rats with PL lesions, the number of recruited clusters was reduced. **F,** The feature tuning mechanism of the best fitting model. For rats learning the 1D tasks, the attention weight for the relevant dimension increased across training, whereas the attention weight for the irrelevant dimension decreased across training. This differentiation was impaired for rats with PL lesions. For rats learning the 2D tasks, the attention weights were equivalent between dimensions and across training. This was true for both control and lesioned rats.

SUSTAIN assumes that similar training experiences cluster together in memory (Love et al., 2004). Categories are represented by one or multiple clusters; each cluster reflects a learned group of similar training experiences and is stored in a hidden layer (Fig. 7A; the cluster layer). On each learning trial, the current stimulus is compared to existing clusters, and each cluster is activated according to its similarity to the stimulus. SUSTAIN’s attention mechanism modulates the stimulus before entering the cluster layer (Fig. 7A; the feature tuning mechanism). Each stimulus dimension is multiplied by an attention weight. These weights bias the perception of the stimulus according to category-relevant information and affect how clusters are activated. Cluster activations then project to a decision layer, which makes a probabilistic decision regarding the category membership of the stimulus (Fig. 7A; decision layer).

At the beginning of training, the model contains one cluster centered on the first training stimulus, and attention weights are equivalent across all stimulus dimensions. Then, feedback is provided after each trial, and SUSTAIN updates accordingly. First, category representations within the cluster layer update, such that the current trial stimulus is either integrated into an existing cluster or becomes the center of a newly recruited cluster. New clusters are created in response to stimuli that are ‘surprising.’ The decision to recruit a new cluster is initiated if the model incorrectly classifies a stimulus and the cluster activations exceed the value of a threshold parameter, indicating that the model is relatively confident in its choice. The feature tuning mechanism is also updated so that attention is shifted towards category-relevant dimensions. This is controlled by two parameters. First, a selective attention parameter determines the amount of attentional focus that can be applied in the category task. Second, an attention learning rate parameter determines how quickly this attention resource can be shifted towards relevant dimensions.

Love & Gureckis (2007) proposed a framework by which the functions of the PFC map onto elements of the SUSTAIN model. Specifically, they posit that the PFC functions as the feature tuning mechanism and shifts attention towards category-relevant information. Second, the PFC updates category representations by initiating the decision to recruit a new cluster. To test these predictions, we created three experimental manipulations that simulate the effects of the PL lesions. The first two manipulations disrupted the feature tuning mechanism to test whether the PL is critical for shifting attention to relevant dimensions. First, we lesioned the feature tuning mechanism by setting the two parameters that control the feature tuning mechanism (i.e., the selective attention parameter and the attention learning rate parameter) to 0. As a result, the model could not update its attention weights, rendering the model unable to shift attention to category-relevant dimensions. Second, we permutated the attention weights before each trial. With this manipulation, the model could update its attention weights normally; however, on any given trial, attention may be directed towards category-irrelevant information. Therefore, the model could learn to identify relevant information, but its ability to maintain selective attention to that information across trials was impaired. The third manipulation tested the prediction that the PL initiates the decision to recruit a new cluster in response to ‘surprising’ stimuli. This was accomplished by increasing the cluster threshold parameter that determines when a new cluster is recruited.

Using combinations of these manipulations, we generated five versions of SUSTAIN that each simulated how the PL lesions affected category learning (Fig. 7B). We also added a control model that assumed the lesions had no effect on learning. Each model was optimized to the rats’ averaged learning curves using the MATLAB function *fmincon*. Then, Akaike’s Information Criterion (AIC) was calculated for each optimized model to quantify its goodness-of-fit (Akaike, 1974). The model with the smallest AIC value was determined as the model that best fit the behavior. The function(s) of PL can be inferred from these results.

### 2.14 Perceptual recency effect

With the current design, each rat completed a large number of training trials. This afforded us the ability to examine category learning on a trial-by-trial basis. Importantly, this sensitivity was leveraged to further test the prediction that the PFC updates category representations (Love & Gureckis, 2007). We examined the effect of the PL lesions on perceptual recency effects, which characterize how category performance is influenced by the identity of the most recent training exemplar (Jones, Love, & Maddox, 2006). Recency effects suggest that category decisions are biased towards recent exemplars, which would imply that the learner regularly updates category representations. Assuming representational updating is a function of the PFC, we predicted that recency effects are mediated by the PL.

Recency effects often interact with the perceptual similarity between exemplars. For example, performance is facilitated if the exemplar is perceptually similar to the most recent exemplar (Jones et al., 2006). Therefore, we binned the accuracy^1^ of training trials according to the perceived similarity between the current exemplar (*n*) and the most recent exemplar (*n-1*; Nosofsky, 1986). Perceptual similarity between exemplars *i* and *j* was calculated as:

$$s_{\mathrm{ij}}=e^{{-d}_{\mathrm{ij}}}$$

where *d* is the psychological distance between exemplars *i* and *j*. Psychological distance was defined as,

$$d_{\mathrm{ij}}=\sum_{m=1}^{M}w_{m}*\left|x_{i}-x_{j}\right|$$

where *w_m_* was SUSTAIN’s estimated attention weight for dimension *m* on trial *n,* and *x* was the physical value of the exemplar along dimension *m.* Trial effects were isolated by subtracting the binned accuracies by the average of 1,000 permutations where trial order was shuffled. Therefore, positive recency scores indicate increased accuracy due to trial order, negative scores indicate decreased accuracy due to trial order, and 0 indicates no effect of trial order.

## 3 Results

### 3.1 Histological assessment of PL lesions

Representative lesions are shown in Fig. 2. Each lesion was examined under a light microscope to ensure that it was contained within the PL. PL boundaries were determined according to Paxinos & Watson (1998). All lesions were centered within the PL, and the data from all rats were included in all analyses. Along the rostral/caudate axis, all lesions were contained between bregma +4.3 and +2.4. There were no significant differences in lesion size and location between the males and females. The lesions of three rats (one rat learning a 1D task and two rats learning a 2D task) extended dorsally into the cingulate cortex and ventrally into the infralimbic cortex. However, there were no differences in behavior between rats with these lesions and rats with more selective lesions.

### 3.2 PL lesions impair category learning for 1D tasks but not 2D tasks

All rats completed 15 training sessions to learn either a 1D task or a 2D task. We used linear mixed effects models to examine accuracy, the number of correction trials, and the number of perseverative errors across category training (see Materials & Methods). The full models included fixed effects for group, training session, a quadratic function (across sessions), random effects for the intercept, slope, and the quadratic function, and a covariate for sex. For all measures, there was a significant main effect for training session (Fig. 3). Session accuracy increased across training, and the number of correction trials and perseverative errors decreased across training (Accuracy: *t*(27.11) = 5.20, *p* < .001; Correction trials: *t*(27.04) = 5.81, *p* < .001; Perseverative errors: *t*(27.27) = 5.12, *p* < .001). There were no significant differences between male and female rats (Accuracy: *t*(22.01) = -1.64, *p* = .116; Correction trials: *t*(19.25) = 0.67, *p* = .513; Perseverative errors: *t*(29.01) = 0.46, *p* = .649), suggesting that sex did not affect category learning. There were also no significant differences between controls learning the 1D tasks vs. the 2D tasks (Accuracy: *t*(26.55) = 0.05, *p* = .963; Correction trials: *t*(26.78) = 0.04, *p* = .971; Perseverative errors: *t(*27.02) = 0.46, *p* = .647). This replicates our previous work and suggests that rats normally learn 1D tasks and 2D at the same rate (Broschard et al., 2019).

Compared to controls, rats with PL lesions were impaired in learning the 1D tasks. Specifically, accuracy was impaired, and the number of correction trials and perseverative errors were larger (Fig. 3A-F; Accuracy: *t*(27.40) = 2.43, *p* = .022; Correction trials: *t*(27.33) = 2.31, *p* = .028; Perseverative errors: *t*(27.54) = 2.56, *p* = .030). Conversely, PL lesions did not affect category learning for the 2D tasks (Accuracy: *t*(27.01) = 0.62, *p* = .541; Correction trials: *t*(26.94) = 0.21, *p* = .838; Perseverative errors: *t*(26.87) = 0.33, *p* = .742). Together, these results indicate that the PL lesions impaired category learning for the 1D tasks, but not the 2D tasks. The 1D tasks, but not the 2D tasks, involve category-irrelevant information, and therefore encourage a shift in attention to a single stimulus dimension. Therefore, our results suggest that the PL is important for shifting attention towards category-relevant dimensions and away from irrelevant dimensions (i.e., selective attention). Without the PL, attention may be divided between the relevant and irrelevant dimensions. Under this interpretation, the PL lesions did not affect learning the 2D tasks because, without the PL, rats were biased toward deploying the optimal strategy (i.e., divided attention) as both dimensions were relevant.

### 3.3 Rats with PL lesions learning 1D tasks require more time to categorize exemplars

Next, we examined the amount of time to evaluate each stimulus (Cue RT) and to execute a category decision (Choice RT) using linear mixed effects models (fixed effects: group, training session, a quadratic function (across sessions); random effects: intercept, slope, and the quadratic function; covariate: sex). There were significant main effects of training session for both Cue RT and Choice RT, such that reaction time decreased across training (Fig. 4; Cue RT: *t*(26.31) = 3.47, *p* = .002; Choice RT: *t*(27.02) = 2.51, *p* = .018). There was no significant difference between male and female rats (Cue RT: *t*(37.89) = 0.62, *p* = .538; Choice RT: *t*(28.78) = -0.36, *p* = .720). For controls, Cue RT and Choice RT were not significantly different between rats learning the 1D tasks and the 2D tasks (Cue RT: *t*(26.96) = 2.09, *p* = .045; Choice RT: *t*(27.00) = 0.26, *p* = .796). For rats with PL lesions, Cue RT was significantly larger than the controls for rats learning the 1D tasks (Fig. 4A-B; *t*(27.02) = 3.92, *p* < .001; Fig. 3C), but not the 2D tasks (*t*(26.97) = 1.25, *p* = .223). However, there were no significant group differences in Choice RT (Fig. 4C-D; 1D tasks: *t*(27.04) = 1.55, *p* = .133; 2D tasks: *t*(26.89) = 0.99, *p* = .329). Together, these results suggest that the rats with PL lesions learning the 1D tasks required more time to evaluate each stimulus. However, there were no significant differences in the amount of time to execute a category decision. These results are task-specific, which suggests that this impairment is a consequence of the 1D tasks having both relevant and irrelevant stimulus information.

### 3.4 PL lesions impair choice confidence for rats learning 1D tasks but not 2D tasks

We then examined the effect of PL lesions on touch separation, a measure of choice confidence during the Cue phase (see Material and Methods). A linear mixed effects model (fixed effects: group, training session, a quadratic function across sessions; random effects: intercept, slope, the quadratic function; covariate: sex) examined touch separation for the third touch across training sessions. First, there was a main effect of training session, such that touch separation increased across sessions (Fig. 4; *t*(27.02) = 4.71, *p* < .001). There was no significant difference in touch separation between male and female rats (*t*(26.16) = -0.93, *p* = .360) as well as controls learning the 1D tasks and 2D tasks (*t*(26.95) = 0.30, *p* = .840). For rats with PL lesions, touch separation was impaired for the rats learning the 1D tasks (Fig. 4E; *t*(27.38) = 2.82, *p* = .009), but not 2D tasks (Fig. 4F; *t*(26.96) = 0.53, *p* = .601). These results support the role of PL in learning 1D tasks and suggests that these rats were less confident in their category decisions.

### 3.5 PL lesions impair category generalization for 1D tasks but not 2D tasks

After category training, each rat was presented with five testing sessions to examine category generalization. Testing distributions had identical category means as the training distributions but had increased variance along the relevant dimension (or relevant axis for the 2D tasks) to sample from novel portions of the stimulus space (Fig. 5A). We segregated the testing distributions into three trial types: stimuli that overlapped with the training distributions (Trained), novel stimuli farther from the category boundary (Distal), and novel stimuli closer to the category boundary (Proximal).

Linear mixed effects models (fixed effects: group, trial type, a quadratic function; random effects: intercept, slope, and the quadratic function; covariate: sex) examined accuracy, Cue RT, Choice RT, and touch separation during testing sessions. Generally, performance was poorer for Proximal stimuli compared to Trained stimuli, suggesting that the rats perceived stimuli closer to the category boundary as more difficult (Broschard et al., 2019). Specifically, accuracy and touch separation for Proximal stimuli were significantly lower than Trained stimuli, and Choice RT for Proximal stimuli was significantly larger than Trained stimuli (accuracy: *t*(52) = 8.22, *p* < .001; touch separation: *t*(52) = 2.49, *p* = .016; Choice RT: *t*(52) = 2.76, *p* = .008). Cue RT did not differ significantly between Proximal stimuli and Trained stimuli (*t*(52) = 2.0, *p* = .057). Conversely, rats could easily generalize to the Distal stimuli, and there were no significant differences between Distal stimuli and Trained stimuli (accuracy: *t*(52) = 1.96, *p* = .055; Cue RT: *t*(52) = 0.94, *p* = .353; Choice RT: *t*(52) = 0.85, *p* = .400; touch separation: *t*(52) = 0.89, *p* = .377). Finally, there were no significant differences in all dependent measures between controls that learned the 1D tasks and 2D tasks (Fig. 5B-E; Accuracy: *t*(26) = 0.77, *p* = .448; Cue RT: *t*(31.08) = 0.73, *p* = .470; Choice RT: *t*(30.23) = 0.33, *p* = .747; touch separation: *t*(38.54) = 0.04, *p* = .966).

PL lesions impaired accuracy and touch separation for rats that learned the 1D tasks (Fig. 5B,E; accuracy: *t*(26) = 2.51, *p* = .019; touch separation: *t*(38.54) = 2.95, *p* = .039), but not the 2D tasks (accuracy: *t*(26) = 0.43, *p* = .667; touch separation: *t*(38.54) = 0.41, *p* = .684). Furthermore, Cue RT was significantly larger for rats with PL lesions that learned the 1D tasks, but not the 2D tasks (Fig. 5C; *t*(31.08) = 2.61, *p* = .014; *t*(31.08) = 0.72, *p* = .480, respectively). PL lesions did not affect Choice RT (Fig. 5D; 1D tasks: *t*(30.23) = 0.27, *p* = .787; 2D tasks: *t*(30.23) = 0.97, *p* = .341). There were no significant interactions between trial types (all *p*s > 0.05). There also were no significant differences between male and female rats (all *p* > .05). Together, these results are consistent with the results from training. PL lesions impaired category generalization for rats that learned the 1D tasks, but not the rats that learned the 2D tasks. Rats with PL lesions learning the 1D tasks had lower accuracy, required more time to categorize each stimulus, and had less confidence in their category decisions.

### 3.6 Simple discrimination

After category generalization, rats were trained to learn a control discrimination task. The trial sequence was identical to category training, except only two objects were presented (instead of categories of stimuli; Fig. 6A). This procedure was added to ensure the PL lesions did not cause general deficits that were not specific to categorization (i.e., motivational, perceptual, motor, etc.). Using a 2x2 between ANOVA, there were no significant differences in the number of sessions to reach the learning criterion across groups (Fig. 6B; *F*(3,25) = 0.37, *p* > .05). These results support the conclusion that the observed impairments were specific to categorization.

### 3.7 SUSTAIN modeling: PL affects selective attention and category representations

Using the neural network SUSTAIN, we created three manipulations that simulated potential functions of the PL (Love & Gureckis, 2007). Two of these manipulations disrupted SUSTAIN’s feature tuning mechanism, which learns to shift attention to category-relevant dimensions. These included 1) lesioning the feature tuning mechanism so that attention weights are static across training and 2) shuffling the attention weights before each trial so that attention was not consistently directed towards category-relevant dimensions. The third manipulation tested the prediction that PL lesions limited the ability to recruit new clusters; this was modeled by increasing a cluster recruitment threshold parameter. Five models were created using combinations of these manipulations (Fig. 7B & 7D). Each model was fit to the averaged group data (Fig. 7B & 7D). These models were compared to a control model that assumed the lesions had no effect on learning. The rats’ behavior was best explained when we shuffled the attention weights before each trial and increased the cluster recruitment threshold for the lesion groups (Fig. 7C; Model 5). These results suggest that the PL is important for maintaining attention to category-relevant dimensions as well as building category representations. All models produced a better fit than the control model that assumed the lesions had no effect on learning.

We then examined the best fitting model in Fig. 7D (Model 5) to ascertain how the lesions affected the cluster representations. Fig. 7E shows that, for the controls, SUSTAIN recruited two clusters (one per category) to learn the 1D tasks, but multiple clusters (∼3–5 per category) to learn the 2D tasks (Broschard et al., 2020). These results suggest that 1D categories are normally represented by single prototypes, whereas 2D categories are normally represented by multiple exemplars (Posner and Keele, 1968, Nosofsky, 1986, respectively). Rats with PL lesions recruited fewer clusters compared to controls to learn the 2D tasks, a direct consequence of increasing the cluster recruitment threshold. These results imply that rats with PL lesions learning the 2D tasks may have had sparser category representations compared to controls, even if performance was intact across training (Fig. 7E).

We then examined the feature tuning mechanism of the best-fitting model to characterize how the PL lesions affected selective attention. Fig. 7F demonstrates that 1D tasks were learned by incrementally shifting attention towards the category-relevant dimension (Broschard et al., 2020). Specifically, the attention weight of the category-relevant dimension increased across training trials, whereas the attention weight to the category-irrelevant dimension decreased across training trials. Importantly, this differentiation was much slower and reached lower levels for rats with PL lesions (Fig. 7F). This finding verifies that shuffling the attention weights across trials reduced selective attention by impairing the model’s ability to maintain attention to the relevant dimension. Conversely, the 2D tasks were learned by dividing attention between stimulus dimensions (Fig. 7F; Broschard et al., 2020). The attention weights for both dimensions were equivalent across training, a pattern that was consistent for both controls and rats with PL lesions.

### 3.8 PL lesions impair perceptual recency effects

SUSTAIN was best fit to the averaged group data when it was assumed that the PL lesions reduced the ability to update category representations. Here, we tested this prediction further by examining category learning on a trial-by-trial basis. We predicted that if the PL is critical for updating representations, then the PL lesions should also impair perceptual recency effects, where the learner biases category decisions according to recent training experiences. To test this, we binned the accuracy of training trials according to the perceived similarity between the current exemplar and the most recent exemplar (see Materials and Methods). Then, we subtracted the binned accuracies from iterations where trial order was randomized. Positive recency scores indicate that accuracy was facilitated because of trial order, negative scores indicate that accuracy was impaired because of trial order, and 0 indicates that trial order had no effect on category accuracy.

**Fig. 8:**
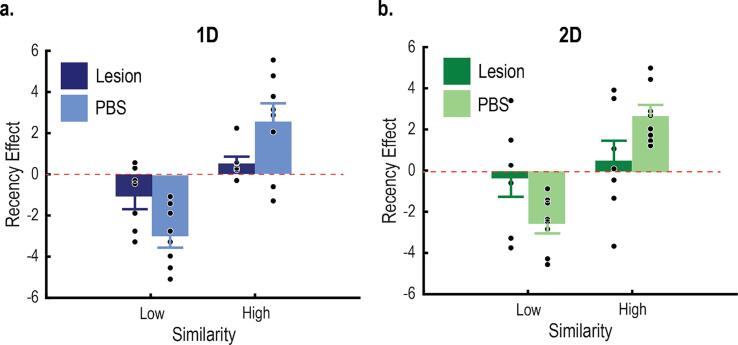
Perceptual recency effects. Accuracy was binned according to the perceptual similarity between the current exemplar and the most recent exemplar. Then, these binned accuracies were subtracted from iterations where trial order was randomized. For controls learning both task types, accuracy was facilitated if the current stimulus had high perceptual similarity to the previous trial (i.e., a positive recency score). Accuracy was impaired if the current stimulus had low perceptual similarity to the previous trial (i.e., a negative recency score). These effects of trial order were absent in rats with PL lesions. This was true for rats learning the 1D (A) and 2D tasks (B). All error bars indicate the *SEM*.

For controls, trial order affected category learning and was modulated by stimulus similarity (Fig. 8). One-sample *t-tests* were used to assess whether the perceptual recency scores were significantly different from 0. For controls learning the 1D and 2D tasks, scores were significantly larger than 0 if the current stimulus was perceptually similar to the previous stimulus (i.e., above the median similarity; 1D tasks: *t*(7) = 3.16, *p* = .016; 2D tasks: *t*(7) = 2.86, *p* = .024). Conversely, scores were significantly smaller than 0 if the current stimulus was perceptually dissimilar from the previous stimulus (i.e., below the median similarity; 1D tasks: *t*(7) = 2.97, *p* = .021; 2D tasks: *t*(7) = 3.01, *p* = .020). These results indicate that accuracy was facilitated if the current stimulus was perceptually similar to the most recent exemplar, but accuracy was impaired if the current stimulus was perceptually dissimilar from the most recent exemplar. For rats with PL lesions, none of the perceptual recency scores were significantly different from 0, indicating that trial order did not affect accuracy (Fig. 8; all *p* > .05). Together, these results indicate that rats normally bias their decisions according to recent training experiences, which implies that they regularly update category representations. This process is effectively absent in rats with PL lesions. This finding supports the SUSTAIN modeling and indicates that the PL is critical for updating category representations.

## 4 Discussion

Rats were trained to categorize stimuli containing black and white gratings according to one stimulus dimension (1D tasks) or two dimensions (2D tasks). Lesions of the PL impaired learning and generalization in rats trained on the 1D tasks. Without the PL, rats learning the 1D tasks had lower accuracy, a larger number of correction trials, and more perseverative errors compared to controls (Fig. 3); they also needed more time to categorize each stimulus (i.e., Cue RT) and showed impaired choice confidence (i.e., touch separation; Fig. 4). The PL lesions did not affect performance on the 2D tasks or the simple discrimination task; therefore, impairments were specific to the 1D tasks. 1D and 2D tasks only differed in a simple rotation of the category distributions. This rotation did not change any physical properties of the categories (Ashby, Smith, & Rosedahl, 2019; e.g., discriminability, average category distance, etc.), but it did affect how the tasks were learned by changing the number of category-relevant dimensions.

COVIS posits that humans have a PFC-mediated declarative system that learns new categories by testing rules (Ashby et al., 1998, Ashby and Maddox, 2011). This system is biased towards simple rules; therefore, COVIS predicts that the PFC is critical for learning tasks that can be solved by unidimensional strategies (i.e., 1D tasks, but not 2D tasks). Using this logic, we could conclude that rats also have a PFC-mediated declarative system that is important for learning 1D tasks. However, there is little evidence that rats consistently apply category rules in the manner that humans do (Broschard et al., 2019). Rule-based learning in humans is best characterized by a step-wise learning curve, where accuracy improves rapidly in a non-linear way (Ashby & Maddox, 2011). Presumably, this jump in performance is a consequence of the participant testing hypotheses about potential rules and selecting the correct rule. Category learning in rats is generally linear and incremental, even for the 1D tasks, suggesting that rats are not testing hypotheses in the same way.

Instead, we propose that rodent PL mediates lower-level mechanisms that make up the building blocks of the primate declarative system. Specifically, the rodent PL biases attention to relevant stimulus information, a mechanism important for learning 1D tasks, but not for learning 2D tasks. This interpretation is supported by SUSTAIN. The neural network model best fit the PL lesion data when we shuffled the attention weights before each decision, suggesting that the PL normally maintains attention to relevant stimulus information (Fig. 7). Shuffling the attention weights did not affect performance on the 2D tasks since attention was allocated to both dimensions equally. This interpretation converges with multiple studies implicating the PL in selective attention by orienting attention to cues that predict reward (Sharpe and Killcross, 2015, Sharpe and Killcross, 2018, Tait et al., 2014).

Selective attention is foundational to categorization (Nosofsky, 1986). At its core, category learning involves discriminating between relevant and irrelevant stimulus information. To illustrate this point, Rehder & Hoffman (2005) tracked eye movements while participants learned to categorize stimuli made from three binary dimensions; depending on the task, the number of category-relevant dimension(s) differed (Shepard, Hovland, & Jenkins, 1961). Eye fixations (and presumably attention) were initially distributed across all stimulus dimensions, but then became restricted to only the relevant dimensions (Rehder & Hoffman, 2005). Our results suggest that maintaining attention to a subset of stimulus dimensions is mediated by the PL, a function that becomes more critical as the number of relevant dimensions decreases. This interpretation also matches the results of Mack et al. (2020), who found that BOLD activity in the ventromedial PFC (vmPFC) tracked the number of relevant stimulus dimensions. They argued that the that vmPFC was critical for filtering out irrelevant stimulus information.

Future experiments can investigate whether other prefrontal subregions are also necessary for learning 1D tasks. A potential target is the anterior cingulate cortex (ACC), which has also been implicated in selective attention in rats (Kim, Wasserman, Castro, & Freeman, 2016). COVIS posits that the ACC participates in the declarative system by switching attention to alternative category rules (Ashby et al., 1998). This can be tested directly by inactivating the rodent ACC before category training. One interesting prediction would be that the PL and ACC serve similar but dissociable functions in selective attention. For example, whereas our results suggest that the PL is critical for maintaining attention to relevant dimensions, the ACC may be critical for identifying dimensions that are category-relevant vs. irrelevant. In this example, the ACC would be critical for learning how to orient attention, and the PL would be critical in applying those learned attention weights.

In addition to selective attention, the results from the SUSTAIN modeling suggest that the PL is also important for creating new category representations (i.e., clusters). SUSTAIN recruits new clusters in response to ‘surprising’ stimuli, where the model is confident in an ultimately incorrect decision (Love et al., 2004). In the current experiment, SUSTAIN best fit the learning data when it was assumed that the rats with PL lesions had a higher threshold to recruit new clusters (Fig. 7). Consequently, without the PL, the category representations were much sparser. This was especially critical for rats learning the 2D tasks, where normally multiple clusters are recruited for each category. The role of the PL in updating category representations was also examined by analyzing category learning on a trial-by-trial basis (Fig. 8). We found that, for controls, category decisions were directly influenced by recent exemplars. Accuracy was facilitated if the current stimulus was perceptually similar to the previous exemplar, whereas accuracy was impaired if the current stimulus was dissimilar to the previous exemplar, suggesting that rats update category decisions regularly and bias their decisions according to recent information. Importantly, rats with PL lesions showed no effects of trial order. Without the PL, rats may be less sensitive to local changes within the category, which could lead to perseveration in the event of a task switch.

We predict that the role of the PL in updating representations is related to the literature that credits the PFC in the development and maintenance of schemas, which are hierarchical representations of information that help organize memories (Koscik & Tranel, 2012). Schemas extrapolate common elements from distinct episodes (Morton et al., 2017, Pudhiyidath et al., 2019) and rely on an interaction between the PFC and hippocampus (Zeithamova et al., 2012, Schlichting and Preston, 2016). We predict that the PL uses these mechanisms in our categorization tasks to update and elaborate category representations. Indeed, a growing literature suggests that the hippocampus stores category representations that are similar to the clusters described by SUSTAIN (Theves et al., 2020, Mack et al., 2016, Mack et al., 2018). For example, Mok and Love (2019) was able to fit a clustering model to the neural activity of place cells and grids cells as a rat navigated an environment. This implies that updating and building category representations involves a close interaction between the PL and hippocampus. Future experiments can examine this interaction directly.

Finally, it is important to note that although the PL facilitates category learning, it may not be necessary for categorization to occur. Indeed, accuracy impairments in the 1D tasks largely occurred during the initial training sessions, and rats with PL lesions were able to learn the 1D tasks after extensive training. This implies that other neural regions were able to compensate. COVIS predicts that a second learning system, the non-declarative system, takes over when the PFC-mediated declarative system cannot successfully find a category rule (Ashby et al., 1998, Ashby and Maddox, 2011). Importantly, key features of this non-declarative system were present in rats with PL lesions. For instance, the non-declarative system does not employ executive functions like selective attention. Additionally, learning in the non-declarative system is thought to be more static and habitual, relying on repetition and consistent feedback. We suspect that in the absence of the PL, a learning system synonymous to the non-declarative system of COVIS compensated. We hypothesize that the dorsolateral striatum (the tail of the caudate nucleus in primates) supports categorization in the absence of the PL, as this region is important for supporting habitual behaviors in rats (Balleine, Delgado & Hikosaka, 2007).

To conclude, a general function of the PFC is to guide behaviors in an adaptive way (Miller & Cohen, 2001). In the context of category learning, we conclude that the rodent PL accomplishes this function through two mechanisms. First, the PL maintains attention to relevant stimulus information (i.e., selective attention); this prevents the incorporation of irrelevant information into category decisions. Second, the PL regularly updates category representations and biases decisions according to recent information; this allows for dense, flexible representations and primes the organism for changes in the category structure. Together, these mechanisms allow for category representations that are both flexible and adaptive.

## Funding

10.13039/100000002National Institutes of Health Grant P01-HD080679 to J.H.F. and B.C.L. and 10.13039/100010269Wellcome Trust Investigator Award WT106931MA to B.C.L.

## Declaration of Competing Interest

The authors declare that they have no known competing financial interests or personal relationships that could have appeared to influence the work reported in this paper.
